# Musculocontractural Ehlers-Danlos Syndrome Leading to Hemorrhagic Shock From Giant Subcutaneous Hematoma: A Case Report

**DOI:** 10.7759/cureus.66774

**Published:** 2024-08-13

**Authors:** Yoshiki Uemura, Norihiko Tsuboi, Satoshi Nakagawa

**Affiliations:** 1 Department of Critical Care Medicine, National Center for Child Health and Development, Tokyo, JPN

**Keywords:** trauma pediatric, chst14, carbohydrate sulfotransferase-14, dermatan sulfate epimerase, musculocontractural ehlers–danlos syndome

## Abstract

The patient was a six-year-old boy with a history of musculocontractural Ehlers-Danlos syndrome (mcEDS). He presented to the emergency department after falling on the road the day before admission, which led to an increase in subcutaneous hematoma in his left lower leg and brief syncope. Initial blood tests revealed a decreased hemoglobin level of 8.1 g/dL (normal range: 14 g/dL). Contrast-enhanced CT showed a massive subcutaneous and intermuscular hematoma in the left thigh. He was diagnosed with hemorrhagic shock due to this extensive hemorrhage and was admitted to the ICU. The affected area was elevated, and hemostasis was achieved through compression. The swelling gradually improved, and he was discharged from the hospital on day 13 after admission. EDS is a systemic condition caused by genetic mutations affecting collagen and collagen-modifying enzymes. mcEDS is an extremely rare variant with a recently identified causative gene, characterized by abnormal connective tissue development and progressive fragility. Giant subcutaneous hematomas resulting from tissue fragility are serious complications of this disease, often occurring with minor trauma and sometimes leading to gradual hemorrhagic shock. Desmopressin nasal drops can be effective in preventing such hematomas. It is crucial to consider the risk of hemorrhagic shock from subcutaneous hemorrhage in patients with mcEDS, especially when repeated subcutaneous hematomas of unknown origin are observed.

## Introduction

Ehlers-Danlos syndrome (EDS) is a systemic disorder resulting from genetic mutations in collagen and collagen-modifying enzymes, with varying clinical presentations depending on the subtype. It has an overall prevalence of 1 in 5,000 individuals [1]. Musculocontractural EDS (mcEDS) is an extremely rare form, with the causative genes recently identified as carbohydrate sulfotransferase 14 (CHST14) or dermatan sulfate epimerase (DSE). This subtype is characterized by abnormal connective tissue development and progressive fragility, leading to complications such as pneumothorax, pneumohemothorax, recurrent joint dislocations, and giant subcutaneous hematomas - features not always present in other EDS subtypes [2]. Giant subcutaneous hematomas resulting from tissue fragility are among the most severe complications of mcEDS [3]. We present a case of a patient with mcEDS who developed hemorrhagic shock due to a massive hematoma following minor trauma.

## Case presentation

**Figure 1:**
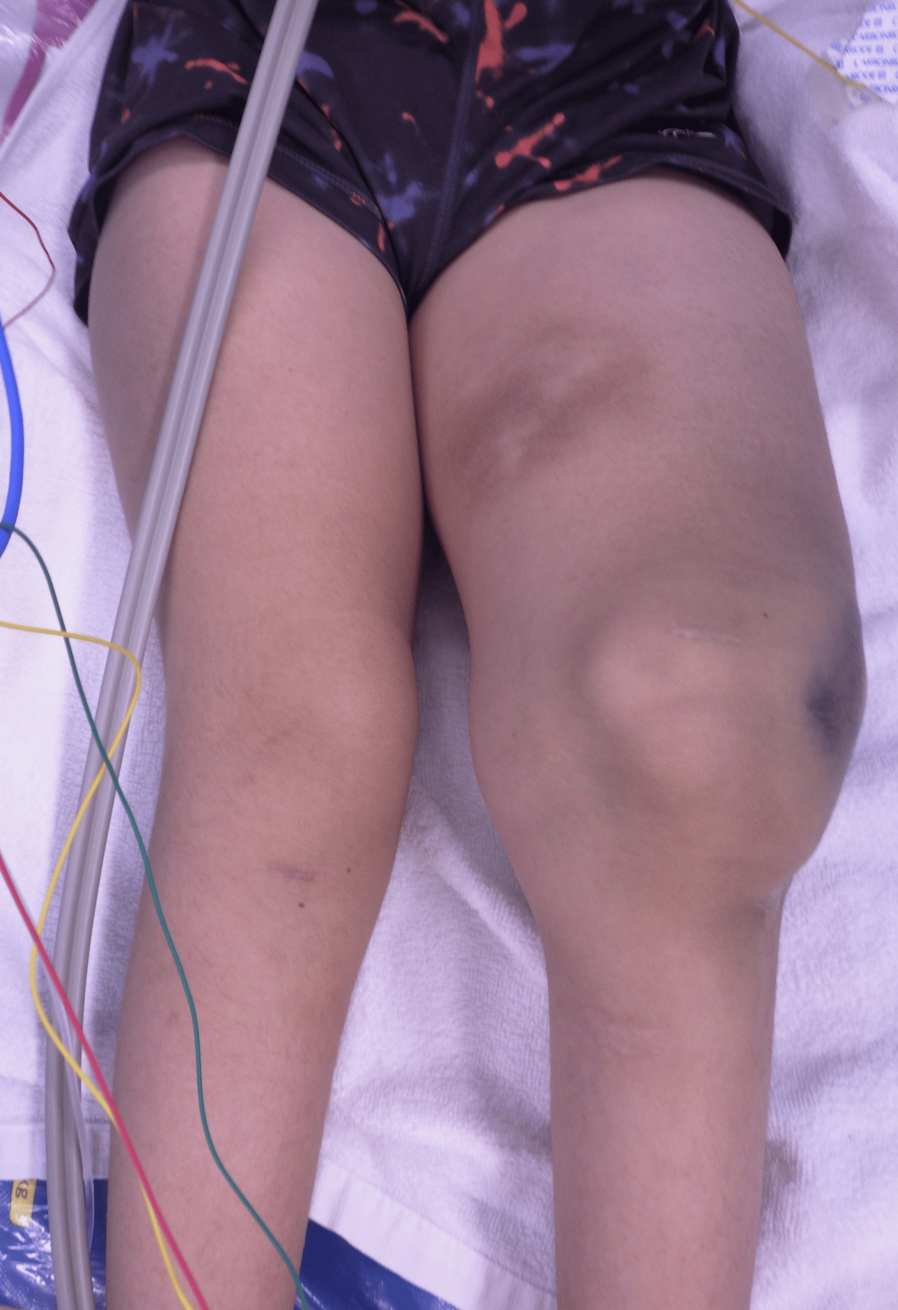
Internal hemorrhage and significant swelling of the left thigh The circumference of the left thigh was 47 cm, compared to 36 cm for the right thigh.

**Figure 2:**
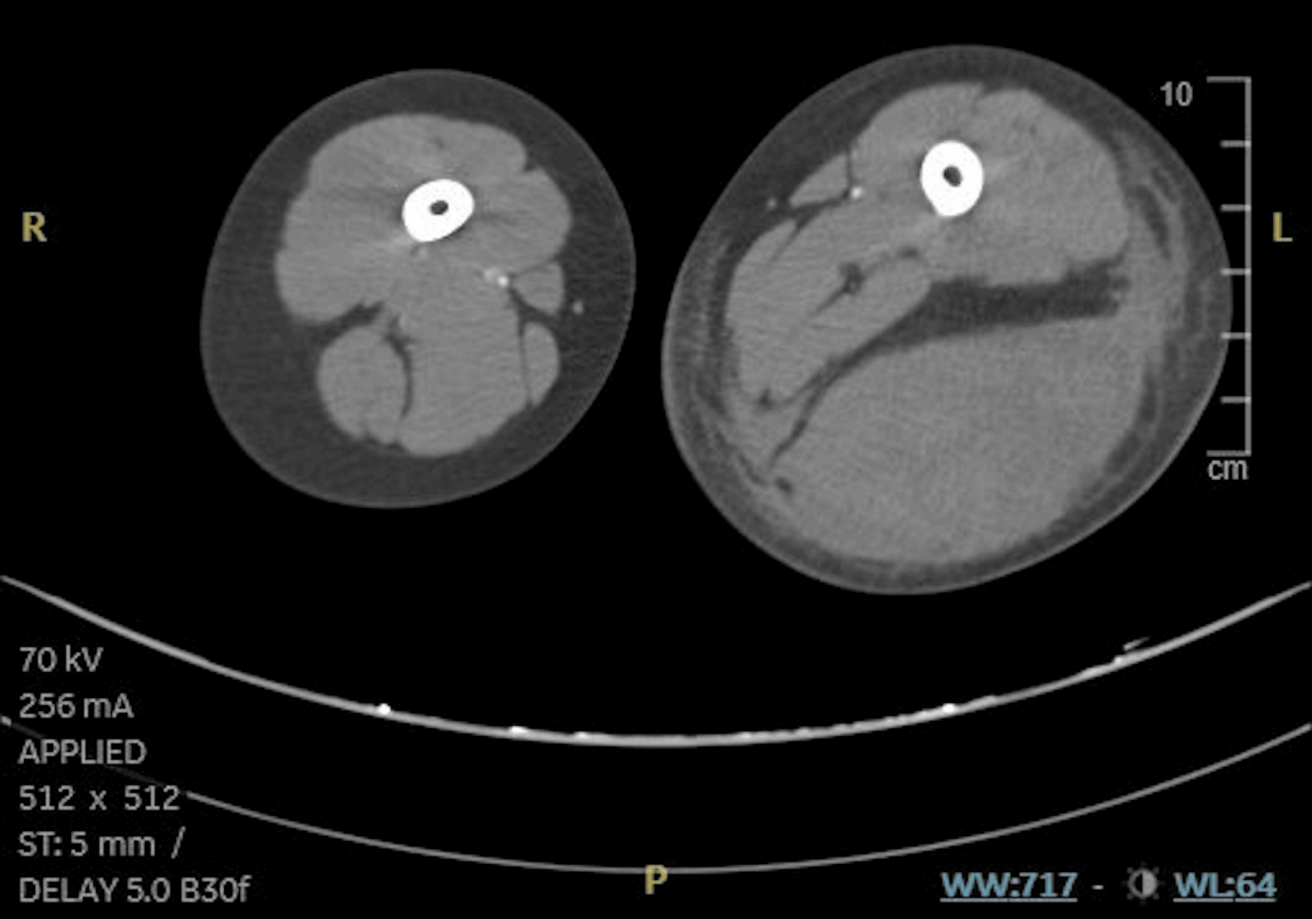
CT showing prominent subcutaneous and intermuscular hematoma in the left thigh

The patient was a six-year-old boy with a history of mcEDS caused by CHST14. Born with multiple joint contractures, he was diagnosed with mcEDS by genetic testing at the age of 3. After falling on the road and bruising his left thigh, swelling and pain in the left thigh gradually worsened. The day following the injury, he was found pale and unconscious for several seconds and was taken to the emergency room. At the time of consultation, his consciousness was clear, his heart rate was 160 beats/min, his blood pressure was 131/77 mmHg, and internal hemorrhage with marked swelling of the left thigh was observed (Figure 1). Blood tests showed decreased hemoglobin at 8.1 g/dL (normal: 14 g/dL), but coagulation function values were normal and bleeding time was within the normal range. Contrast-enhanced CT revealed no obvious arterial bleeding but showed a massive subcutaneous and intermuscular hematoma in the left thigh (Figure 2). There was no significant hemorrhage at other sites. Hemorrhagic shock from subcutaneous massive hemorrhage was diagnosed, and the patient was admitted to the intensive care unit. Tachycardia improved with crystalloid solution administration. Hemoglobin dropped to 6.9 g/dL over time, and tranexamic acid was administered to halt bleeding progression. The affected area was elevated, and hemostasis was achieved by compression. During management, careful observation was made to ensure that compression did not cause additional hemorrhage or disrupt tissue perfusion in the lower leg. The swelling gradually improved, and he was discharged from the hospital on day 13 after admission.

## Discussion

mcEDS is an exceedingly rare subtype of EDS, with a prevalence of approximately 1 in 1 million. Recent advances have identified the causative genes for mcEDS as CHST14 or DSE. This condition is marked by abnormal development, such as distinctive facial features and congenital joint contractures, and progressive connective tissue fragility, which can manifest as skin hyperextension, systemic joint relaxation, chronic dislocations, and giant subcutaneous hematomas [2]. mcEDS-CHST14 tends to present more frequently with symptom crises compared to mcEDS-DSE [4]. The fragility observed in connective tissues is attributed to collagen fibril assembly failures linked to systemic deficiencies in dermatan sulfate [3]. Early diagnosis of mcEDS should be considered in patients exhibiting these symptoms, with genetic testing being crucial for confirmation. Giant subcutaneous hematoma, a severe complication of this disease, can arise from minor trauma and may progress gradually to cause hemorrhagic shock [5]. Studies indicate that 80% of patients with large subcutaneous hematomas experience their first episode by the age of 12 [6]. The prognosis for mcEDS remains uncertain due to the limited number of cases. Currently, no curative treatment exists for mcEDS. However, preventive measures can be employed for all EDS forms. Desmopressin nasal drops have proven effective in preventing subcutaneous hematomas [7]. Previous reports have shown that DDAVP treatment following trauma can avert the development of large hematomas in mcEDS patients [3]. Desmopressin is believed to improve platelet-collagen interactions impaired by EDS and enhance von Willebrand factor-mediated platelet adhesion [8]. Additionally, tranexamic acid may help halt bleeding progression and can be administered in primary care settings [9].

## Conclusions

In the present case, hemostasis was delayed due to the spread of venous hemorrhage within the fragile subcutaneous tissue and muscle, triggered by a minor contusion. This led to hemorrhagic shock. It is crucial to be aware of the risk of hemorrhagic shock from subcutaneous hemorrhage in patients with mcEDS. Repeated subcutaneous hematomas of unknown origin should prompt suspicion of this disease. Early genetic testing and diagnosis are essential for timely preventive measures, which can significantly improve the prognosis for patients with mcEDS.

## References

[1] Beighton P, De Paepe A, Steinmann B (1998). Ehlers-Danlos syndromes: revised nosology, Villefranche, 1997. Ehlers-Danlos National Foundation (USA) and Ehlers-Danlos Support Group (UK). Am J Med Genet.

[2] Kosho T, Mizumoto S, Watanabe T, Yoshizawa T, Miyake N, Yamada S (2019). Recent advances in the pathophysiology of musculocontractural Ehlers-Danlos syndrome. Genes (Basel).

[3] Kosho T, Miyake N, Hatamochi A (2010). A new Ehlers-Danlos syndrome with craniofacial characteristics, multiple congenital contractures, progressive joint and skin laxity, and multisystem fragility-related manifestations. Am J Med Genet A.

[4] Minatogawa M, Hirose T, Mizumoto S (2022). Clinical and pathophysiological delineation of musculocontractural Ehlers-Danlos syndrome caused by dermatan sulfate epimerase deficiency (mcEDS-DSE): a detailed and comprehensive glycobiological and pathological investigation in a novel patient. Hum Mutat.

[5] Daşar T, Donkervoort S, Şimşek Kiper PÖ (2022). A life-threatening complication in a patient with Ehlers-Danlos syndrome musculocontractural type. J Pediatr Res.

[6] Minatogawa M, Unzaki A, Morisaki H (2022). Clinical and molecular features of 66 patients with musculocontractural Ehlers-Danlos syndrome caused by pathogenic variants in CHST14 (mcEDS-CHST14). J Med Genet.

[7] Mast KJ, Nunes ME, Ruymann FB, Kerlin BA (2009). Desmopressin responsiveness in children with Ehlers-Danlos syndrome associated bleeding symptoms. Br J Haematol.

[8] Mannucci PM (1998). Hemostatic drugs. N Engl J Med.

[9] Angwin C, Doolan BJ, Hausser I (2024). Skin fragility and wound management in Ehlers-Danlos syndrome: a report by the Ehlers Danlos Syndrome society skin working group. Clin Exp Dermatol.

